# Cellular Uptake and Photo-Cytotoxicity of a Gadolinium(III)-DOTA-Naphthalimide Complex “Clicked” to a Lipidated Tat Peptide

**DOI:** 10.3390/molecules21020194

**Published:** 2016-02-05

**Authors:** William I. O’Malley, Riccardo Rubbiani, Margaret L. Aulsebrook, Michael R. Grace, Leone Spiccia, Kellie L. Tuck, Gilles Gasser, Bim Graham

**Affiliations:** 1 Monash Institute of Pharmaceutical Sciences, Monash University, Parkville VIC 3163, Australia; william.o’malley@monash.edu; 2 Department of Chemistry, University of Zurich, Winterthurerstrasse 190, Zurich CH-8057, Switzerland; riccardo.rubbiani@chem.uzh.ch; 3 School of Chemistry, Monash University, Clayton VIC 3800, Australia; margaret.aulsebrook@monash.edu (M.L.A.); mike.grace@monash.edu (M.R.G.); leone.spiccia@monash.edu (L.S.)

**Keywords:** cell-penetrating peptide, fluorescence, gadolinium complex, photo-cytotoxicity, theranostic

## Abstract

A new bifunctional macrocyclic chelator featuring a conjugatable alkynyl-naphthalimide fluorophore pendant group has been prepared and its Gd(III) complex coupled to a cell-penetrating lipidated azido-Tat peptide derivative using Cu(I)-catalysed “click” chemistry. The resulting fluorescent conjugate is able to enter CAL-33 tongue squamous carcinoma cells, as revealed by confocal microscopy, producing a very modest anti-proliferative effect (IC_50_ = 93 µM). Due to the photo-reactivity of the naphthalimide moiety, however, the conjugate’s cytotoxicity is significantly enhanced (IC_50_ = 16 µM) upon brief low-power UV-A irradiation.

## 1. Introduction

There continues to be much interest in the development of molecular imaging agents, including ones that can be detected by more than one imaging modality (“multi-modal imaging agents”) [1,2,3,4,5]. This is particularly so within the field of oncology, where imaging agents are used extensively for the screening, diagnosis, staging, planning of treatment, post-treatment assessment and surveillance of cancer [6,7]. Another area that has seen rapid growth over the last decade is the development of agents that can be used for both imaging and therapy (“theranostics”), allowing for image-guided drug delivery, *in vivo* detection of drug release/activation, and/or monitoring of patient’s response to therapy [8,9,10]. Designs range from small molecules and (bio)conjugates [11] through to multi-functional nanoparticulate systems [12,13].

The most advanced theranostic designs are engineered to be stimuli-responsive, with activation of therapeutic activity/drug release occurring in response to endogenous triggers (e.g., pH change, hypoxia, elevated enzyme activity) or external stimuli (e.g., heat, light). This allows for controlled dosing and/or reduced exposure of non-diseased cells/tissue to cytotoxic species [14,15]. The development of *photo-responsive* systems, in particular, has received considerable attention, since light, as a stimulus, is generally non-invasive and can be readily manipulated, enabling drug activation/release to be controlled both spatially and temporally with extreme precision. Photo-responsiveness is often achieved by loading bioactive cargo into carrier (nano)materials that are amenable to dissociation/structural change upon exposure to light [16,17,18]. Alternatively, light-activated pro-drugs may be employed in the construction of photo-responsive theranostic designs. These include photo-sensitisers, which generate cytotoxic singlet oxygen (^1^O_2_) in response to light and form the basis of photo-dynamic therapy (PDT) [19,20,21], and photo-activated chemotherapeutic (PACT) agents, which induce cell death through mechanisms such as light-mediated ligand ejection, DNA crosslinking and uncaging [22,23,24,25,26,27]. As an added benefit, many PDT and PACT agents are luminescent, providing a ready means of detection [28,29,30].

The development of increasingly elaborate and sophisticated multi-modal imaging agent and theranostic designs, including photo-responsive ones, has been aided by the advent of “bio-orthogonal” chemistries, such as the Cu(I)-catalysed azide-alkyne cycloaddition reaction (“click reaction”) [31,32] and its Cu-free variant—strain-promoted azide-alkyne cycloaddition (SPAAC) [33,34]. These allow for the late-stage introduction of moieties into highly functionalised molecules (small molecules, peptides, proteins) [35,36,37,38] and nanoparticles [39,40,41], as well as the controlled stepwise elaboration of hetero-multifunctional scaffolds [42,43,44], without the need for complex protection group strategies. The widespread adoption of bio-orthogonal labelling technologies in the biological and biomedical sciences has also seen an expanding toolbox of “clickable” compounds (fluorophores, cross-linkers, macrocyclic chelators, *etc.*) become available to researchers interested in multi-functional imaging and therapeutic agents. Indeed, many such “building blocks” are now commercially available.

We have a particular interest in the development of metal complex-based agents for imaging and therapy, including photo-cytotoxic complexes for potential application as new PDT and PACT agents [45,46,47,48,49,50,51,52]. During the course of our work, a number of “clickable” metal complexes/chelators have been developed and employed in the synthesis of peptide conjugates with tumour-targetting, cell-penetrating and/or organelle-specific localising properties [53,54,55,56]. Additionally, we have incorporated alkyne-bearing metal complexes into proteins via click conjugation to unnatural amino acids to facilitate protein structural investigations [57,58,59]. Many other research groups have likewise reported alkyne- and azide-bearing metal complexes/chelators for a range of biological and biomedical applications [60,61,62,63,64].

As part of an effort to generate new photo-activated theranostics and multi-modal imaging agents, we have now developed a new macrocyclic gadolinium(III) complex of a 1,4,7,10-tetraazacyclodocane-1,4,7,10-tetraacetic acid (DOTA) derivative featuring a conjugatable alkynyl-napthalimide pendant. Naphthalimide derivatives are widely employed as fluorescent dyes and are known to be photo-reactive [65,66,67,68,69,70,71], while Gd(III)-DOTA-type complexes are well-established MRI contrast agents [72,73,74]. To demonstrate its utility, the complex has been conjugated to a “model” peptide—a lipidated, azide-bearing derivative of the cell-penetrating “Tat” peptide, derived from the HIV-1 “Trans-Activator of Transcription” protein [75]. We show that the “clicked” naphthalimide moiety can be used for fluorescence tracking and results in a photo-cytotoxic effect when cellular entry of the complex is facilitated by conjugation to the Tat peptide.

## 2. Results and Discussion

### 2.1. Synthesis of Ligand and Gd(III) Complexes

**Scheme 1:**
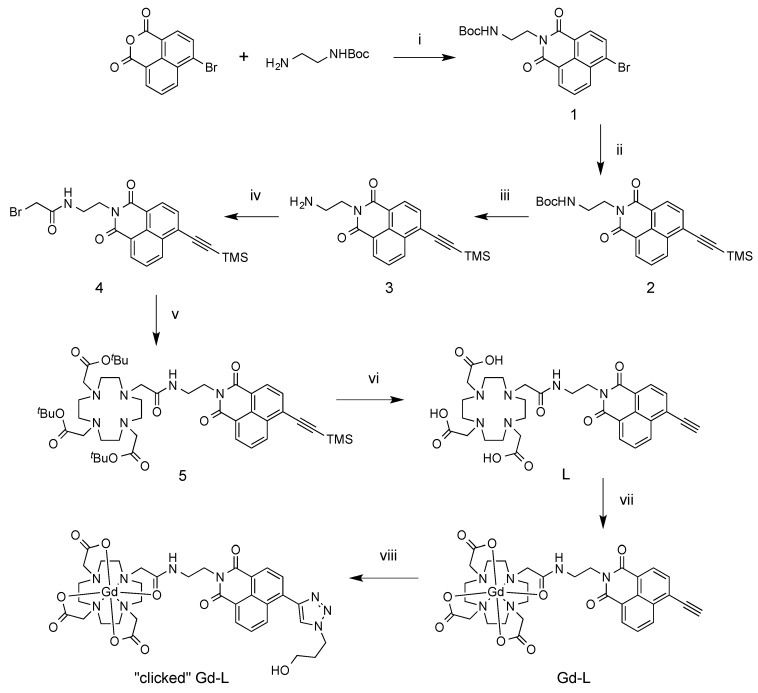
Synthesis of ligand and Gd(III) complexes. (i) DMF, 80 °C, overnight (O/N), 91%; (ii) TMS acetylene, Pd(PPh_3_)_2_Cl_2_, CuI, Et_3_N, THF, N_2_ atmosphere, RT, 3 h, 48%; (iii) TFA, DCM, RT, 4 h, 96%; (iv) bromoacetyl bromide, Na_2_CO_3_, acetone, RT, 3 h, 59%; (v) ^t^Bu_3_DO_3_A.HBr, DIPEA, ACN, reflux, O/N, 81%; (vi) (a) KF, H_2_O/ACN, RT, 2 h; (b) TFA, DCM, RT, O/N, 71% (over two steps); (vii) Gd(OAc)_3_, water/ACN, 70 °C, 2 h, quant. (53% isolated); (viii) 3-azido-1-propanol, CuSO_4_, sodium ascorbate, THPTA, water/ACN, pH 7, RT, O/N, quant. (47% isolated).

The synthesis of the ligand, L, commenced with a condensation reaction between commercially-available 4-bromo-1,8-naphthalic anhydride and *N*-Boc-1,2-ethylenediamine, followed by a Sonogashira reaction of the product (1) with trimethylsilyl (TMS) acetylene to install a TMS-protected alkyne group in place of the bromo substituent (Scheme 1). Following Boc-deprotection of 2 with trifluoroacetic acid (TFA), the exposed amine of 3 was reacted with bromoacetyl bromide to produce the bromoacetamide derivative, 4. Compound 4 was subsequently coupled to *tert*-butyl-protected 1,4,7,10-tetraazacyclododecane-1,4,7-triacetic acid (^t^Bu_3_DO3A) [76]. Lastly, the TMS and ^t^Bu groups were removed using KF and TFA, respectively, to yield L, which was purified by preparative HPLC.

The Gd(III) complex, Gd-L, was prepared by heating a neutral (pH 6.5–7.5) aqueous solution of the ligand with two equiv. of gadolinium(III) acetate for 2 h, after which time LC-MS analysis indicated near-quantitative complexation. Preparative HPLC was then used to purify the complex to >95% purity.

As an initial test, Gd-L was conjugated to 3-azido-1-propanol as a model azide in aqueous solution. CuSO_4_ (0.1 equiv.) was utilised as the copper source, sodium ascorbate (1 equiv.) as the reducing agent and *tris*(3-hydroxypropyl-triazolylmethyl)amine (THTPA) (0.2 equiv.) as a Cu(I)-stabilising ligand [77,78]. After stirring overnight, conversion to the clicked product was near-quantitative according to LC-MS analysis. The complex was again isolated in >95% purity following preparative HPLC.

### 2.2. Photo-Physical Properties of Complexes

**Figure 1:**
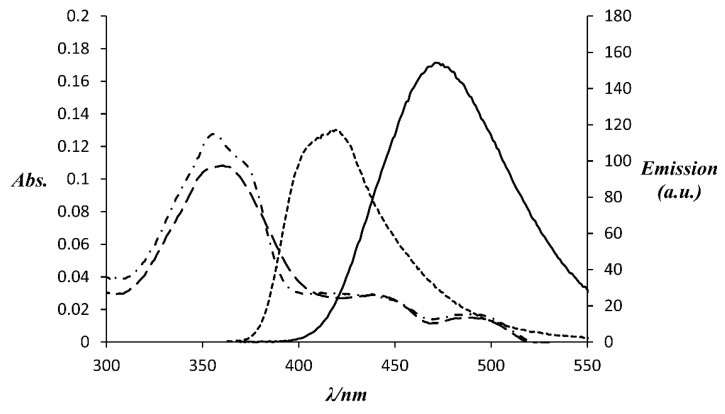
Absorbance and fluorescence emission spectra of Gd(III) complexes (5 µM) in 100 mM HEPES, pH 7.4 (298 K). Dot-dashed line: absorbance of Gd-L, dotted line: emission of Gd-L excited at 356 nm, dashed line: absorbance of “clicked” Gd-L, solid line: emission of “clicked” Gd-L excited at 362 nm.

The spectral properties of the two Gd(III) complexes were measured in aqueous solution buffered at pH 7.4 with 100 mM HEPES. Table 1 summarises the data, while absorbance and fluorescence emission spectra of the complexes are shown in Figure 1.

Click conjugation of the Gd-L complex is associated with a slight bathochromic shift (6 nm) in the absorbance band arising from *π-π* transitions within the naphthalimide group. A much larger shift (54 nm) is observed for the fluorescence emission band and the fluorescence quantum yield is increased by *ca.* 70%. These findings are in accordance with those reported for a simple *N*-ethyl naphthalimide derivative bearing an alkyne at the 8-position [79].

**Table 1:** Photo-physical data for Gd(III) complexes measured in 100 mM HEPES, pH 7.4 (298 K).

Complex	Absorption λ_max_ (ε (M^−1^cm^−1^))	Emission λ_max_	Φ ^a^ (%)	Brightness (ε × Φ/1000 (M^−1^cm^−1^))
Gd-L	356 (22,500)	417	35%	7.9
“Clicked” Gd-L	362 (19,200)	471	59%	11.3

^a^ Quantum yield measured relative to quinine sulphate [80].

### 2.3. Conjugation of Complex to Lipidated Tat Peptide

**Figure 2:**
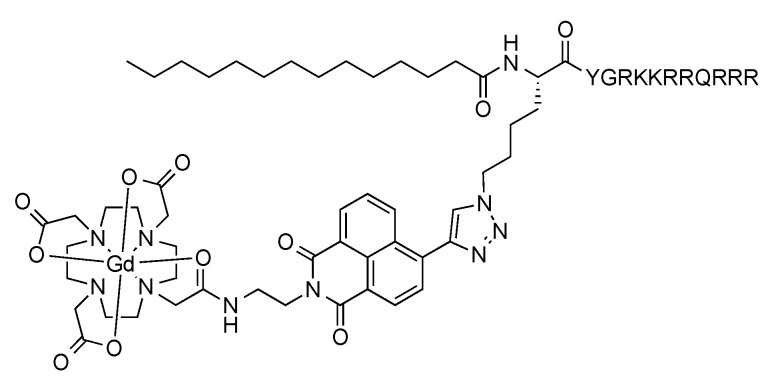
Structure of Gd-L-bearing myristylated Tat peptide conjugate.

To demonstrate its utility, the Gd-L complex was ligated to a derivative of the cell-penetrating Tat peptide featuring a myristic acid tail at its N-terminus (to aid with cellular uptake) and an internal azido-L-lysine residue (Figure 2). We have previously attached a luminescent rhenium(I) complex to this lipidated peptide and visualised uptake of the resulting conjugate into cells via fluorescence microscopy [55]. Stirring the peptide with Gd-L in the presence of CuSO_4_, sodium ascorbate and THPTA at room temperature overnight led to essentially quantitative conversion, as judged by LC-MS analysis. The product was isolated in 90% purity following preparative HPLC.

### 2.4. (Photo-)cytotoxicity of Complexes and Conjugate

**Figure 3:**
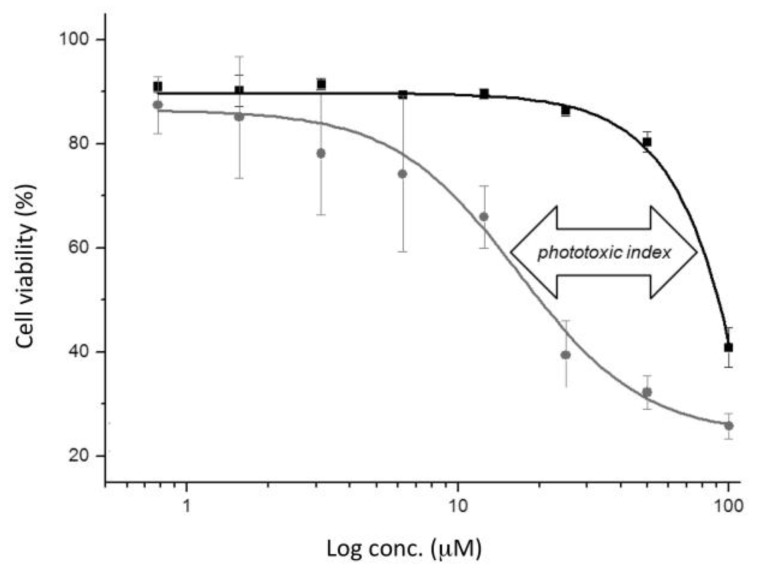
Dose-response curves for the anti-proliferative effect of Gd-L-Tat peptide conjugate on CAL-33 cells in the dark (black line) and upon UV-A irradiation (at 350 nm for 10 min, 2.58 J·cm^−2^; grey line).

The cytotoxic properties of Gd-L, “clicked” Gd-L and the Gd-L-Tat peptide conjugate were assessed on CAL-33 tongue squamous carcinoma cells, either in the dark or combined with short exposure to a low-power dose of UV-A radiation (350 nm, 2.58 J·cm^−2^, 10 min). As the results in Table 2 indicate, the Gd-L and “clicked” Gd-L complexes exerted essentially no cytotoxic effect under either of these conditions, with measured IC_50_ values >100 μM in all cases. The Tat conjugate also produced only a very modest anti-proliferative effect when incubated with cells in the dark (IC_50_ ≈ 93 μM). However, a six-fold enhancement in cytotoxicity (IC_50_ ≈ 16 μM) was observed when the conjugate was exposed to UV-A radiation (see also Figure 3). These results reflect, first of all, that the myristylated Tat peptide aids considerably with cellular uptake [55,81]. Secondly, the observed photo-cytotoxicity of the conjugate is consistent with previous reports of the photo-reactivity of naphthalimide derivatives towards biological molecules, including proteins and nucleic acids [65,66,67,68,69,70,71].

**Table 2:** Anti-proliferative effects of the Gd(III) complexes and Tat peptide conjugate on CAL-33 cancer cells in the dark and upon light irradiation at 350 nm for 10 min (2.58 J·cm^−2^).

Compound	IC_50_ (Dark) ^a^ (μM)	IC_50_ (UV-A) ^a^ (μM)	PI ^b^ (x-Fold)
Gd-L	>100	>100	n.a.
“clicked” Gd-L	>100	>100	n.a.
Gd-L-Tat conjugate	93 ± 3	16 ± 6	5.8

^a^ expressed as mean ± standard error of independent experiments; ^b^ PI = photo-toxic index = IC_50_ (UV-A)/IC_50_ (dark). n.a. = not applicable.

### 2.5. Cellular Uptake of Complexes and Conjugates

**Figure 4:**
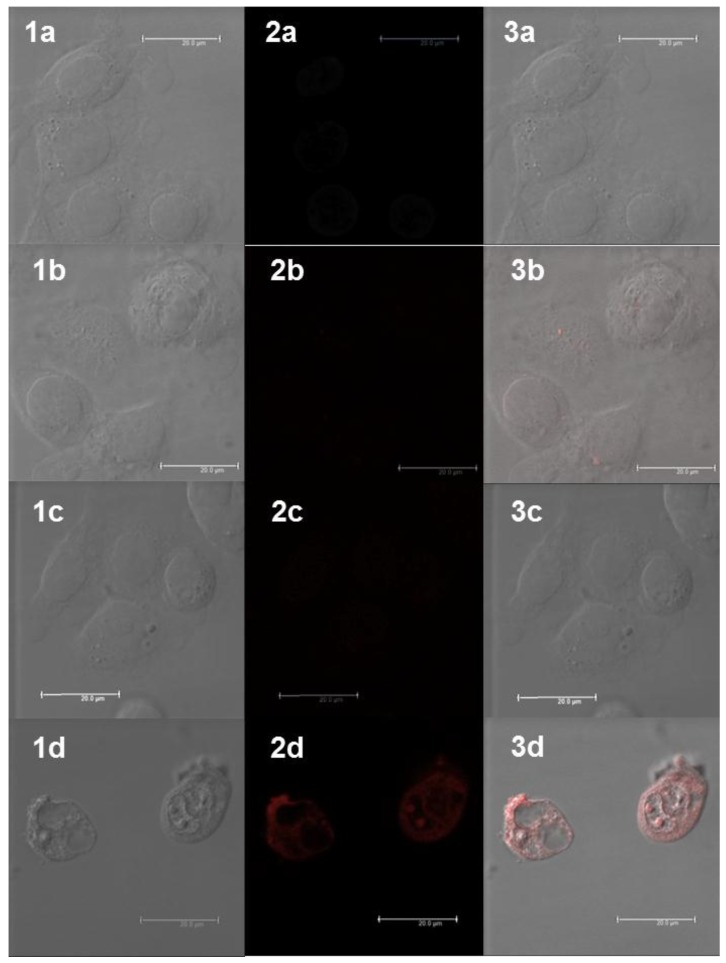
Confocal fluorescence microscopic images of CAL-33 cells incubated with Gd(III) complexes or conjugate for 4 h: (**1a**) DIC (differential interference contrast) image of the untreated Cal-33 cells; (**2a**) visualisation of background fluorescence in untreated CAL-33 cells using the excitation/emission wavelength settings (hybrid 1 channel) employed for detection of the Gd(III) compounds; (**3a**) merged image of the DIC and hybrid 1 channel images of untreated cells; (**1b**) DIC image of the CAL-33 cells treated with 100 μM of “clicked” Gd-L; (**2b**) visualisation of “clicked” Gd-L in cells; (**3b**) merged image of the DIC and hybrid 1 channel images of cells treated with “clicked” Gd-L; (**1c**) DIC image of the CAL-33 cells treated with 100 μM of Gd-L; (**2c**) visualisation of Gd-L in cells; (**3c**) merged image of the DIC and hybrid 1 channel images of cells treated with Gd-L; (**1d**) DIC image of the CAL-33 cells treated with 100 μM of Gd-L-Tat peptide conjugate; (**2d**) visualisation of Gd-L-Tat peptide conjugate in cells; (**3d**) merged image of the DIC and hybrid 1 channel images of cells treated with Gd-L-Tat peptide conjugate. The scale bars represent 20 μm.

The ability of the two Gd(III) complexes and the Tat conjugate to enter CAL-33 cells was assessed using confocal fluorescence microscopy (Figure 4). Although the optimal excitation and emission wavelengths for the naphthalimide fluorophore are 356 and 417 nm for Gd-L and 362 and 471 nm for “clicked” Gd-L, respectively (Table 1), the compounds could also be reliably detected using the microscope’s “hybrid 1 red wavelength” channel (excitation: 405 nm; emission: 600−800 nm) because of the brightness of the naphthalimide fluorophore, combined with the broadness of its absorption and emission peaks. For cells incubated with “clicked” Gd-L, only extremely weak fluorescence was observed (panels 2b and 3b), while cells treated with Gd-L were essentially non-fluorescent (panels 2c and 3c), indicating that these complexes are not able to enter CAL-33 cells. In contrast, for cells incubated with the Gd-L-Tat peptide conjugate there was clear evidence of uptake and localisation within the cytoplasmic regions of the cells (panel 3d). This is consistent with the results of previous studies, which have shown that myristylated Tat is an effective cell-penetrating agent [55,81]. The experiments were performed at 100 µM, a concentration that resulted in a clear Gd-L-Tat signal. At this dose, it was possible to observe the early stages of induced cellular stress, in good agreement with the cytotoxicity investigation. It is worthwhile noting that, besides cytosolic uptake, Gd-L-Tat also displayed some punctate accumulation in regions ascribable to the cell nucleoli (panels 2d and 3d). Overall, the confocal microscopy results help to rationalise the cytotoxicity data: the conjugate is the only compound that exerts a cytotoxic effect because it is the only compound to enter the CAL-33 cells.

## 3. Experimental Section

### 3.1. Materials and Methods

All chemicals were purchased from Sigma-Aldrich Pty (St. Louis, MO, USA), Matrix Scientific (Columbia, SC, USA), or Merck Group Ltd (Darmstadt, Germany) and used without purification. 1,4,7,10-Tetraazacyclododecane-1,4,7-triacetic acid (^t^Bu_3_DO3A.HBr) [76] and *tris*(3-hydroxypropyl-triazolylmethyl)amine (THTPA) [77,78], were synthesised as per reported literature procedures. All solvents were reagent, analytical or HPLC grade.

Flash chromatography was carried out using Merck 38 Silica gel 60, 230–400 mesh ASTM. Thin layer chromatography (TLC) was performed on Merck Silica Gel 60 F254 plates. TLC plates were visualised using a UV lamp at 254 nm or through the use of KMnO_4_ or ninhydrin staining agent.

Elemental analyses were performed by the Campbell Microanalytical Laboratory, Otago, New Zealand.

^1^H and ^13^C nuclear magnetic resonance (NMR) spectra were recorded using an Avance III Nanobay 400 MHz Bruker spectrometer (Billerica, MA, USA) coupled to the BACS 60 automatic sample changer at 400.13 MHz and 100.61 MHz, respectively. Data acquisition and processing was managed using Topspin software package version 3. Additional processing was handled with MestReNova software (PC). Chemical shifts (δ) were measured in parts per million, referenced to an internal standard of residual solvent. Spectroscopic data are given using the following abbreviations: s, singlet; d, doublet; dd, doublet of doublets; ddd, doublet of doublet of doublets; dt, doublet of triplets; t, triplet; m, multiplet; J, coupling constant.

Analytical high-performance liquid chromatography (HPLC) was carried out on an Agilent 1260 series modular HPLC (Santa Clara, CA, USA) equipped with the following modules: G1312B binary pump, G1316A thermostated column compartment equipped with an Agilent Eclipse Plus C18 3.5 µm, 4.6 × 100 mm column and a G1312B diode array detector. The following elution protocol was used: 0–10 min, gradient from 5% solvent B/95% solvent A to 100% solvent B (solvent A = 99.9% H_2_O, 0.1% TFA, and solvent B = 99.9% acetonitrile (ACN), 0.1% TFA, except in the case of the analysis of Gd complexes, where solvent A = 99.9% H_2_O, 0.1% formic acid, and solvent B = 99.9% ACN, 0.1% formic acid); flow rate = 1 mL·min^−1^.

Preparative HPLC purification was carried out on an Agilent 1260 modular Prep HPLC equipped with the following modules: G1361A prep pump, G2260A prep automatic liquid sampler, G1364B fraction collector, G1315D diode array detector, and a Luna C8 5 µm, 100 Å AXIA, 250 × 21.2 mm column. The following elution protocol was used: 0–5 min, 100% solvent C; 5–30 min, gradient from 100% solvent A to 20% solvent C/80% solvent D (solvent C = 99.9% H_2_O, 0.1% formic acid; solvent D = 99.9% ACN, 0.1% formic acid); flow rate = 20 mL·min^−1^.

High-resolution mass spectrometric (HRMS) analyses were performed on a Waters LCT TOF LC-MS mass spectrometer (Milford, MA, USA) coupled to a 2795 Alliance Separations module. All data were acquired and mass corrected via a dual-spray Leucine Enkephaline reference sample. Mass spectra were generated by averaging the scans across each peak and background subtracted of the TIC. Acquisition and analysis were performed using the MassLynx software version 4.1. The mass spectrometer conditions were as follows: electrospray ionisation (ESI) mode, desolvation gas flow of 550 L·h^−1^, desolvation temperature of 250 °C, source temperature of 110 °C, capillary voltage of 2400 V, sample cone voltage of 60 V, scan range acquired between 100–1500 *m*/*z*, one sec scan times and internal reference ions for positive ion mode (Leucine Enkephaline) of 556.2771.

Liquid chromatography-mass spectrometry (LC-MS) was performed using an Agilent 6100 Series Single Quad LC-MS coupled to an Agilent 1200 Series HPLC with the following mass spectrometer conditions: multimode-ESI mode, 300 °C drying gas temperature, 200 °C vaporising temperature, capillary voltage of 2000 V (positive), capillary voltage of 4000 V (negative), scan range between 100–1000 *m*/*z* with an 0.1 s step size and a 10 min acquisition time.

Analysis of the peptide conjugates was performed on a Shimadzu modular LC-MS system (Kyoto, Japan) equipped with the following modules: LC-20AD liquid chromatograph system, SPD-M20A diode array detector, CTO-20A column oven equipped with a Luna 3 micron C8(2) 3 µm, 100 Å, 100 × 2.0 mm column and a LC-MS-2020 system, operating in positive mode with an *m*/*z* scan range of 200–2000.

Absorbance spectra were recorded on a Varian Cary 50 Bio UV-Vis spectrophotometer (Palo Alto, CA, USA) using a 1 cm-path length quartz cuvette. Fluorescence emission spectra were acquired using a Varian Cary Eclipse fluorescence spectrophotometer using a 1 cm-path length quartz cuvette.

### 3.2. Synthetic Procedures

#### 3.2.1. *tert-Butyl 2-(6-bromo-1,3-dioxo-1H-benzo[de]isoquinolin-2(3H)-yl)ethylcarbamate* (**1**)

4-Bromo-1,8-napthalic anhydride (3.00 g, 10.8 mmol) and *N*-(*tert*-butoxycarbonyl)-1,2-ethylenediamine (1.91 g, 11.9 mmol) were dissolved in DMF (30 mL) and stirred at 80 °C overnight (O/N). The solution was then allowed to cool to room temperature (RT), before being poured into ice-cold water to produce a yellow precipitate, which was collected via vacuum filtration, washed several times with cold H_2_O and dried in a vacuum desiccator overnight. Yield: 4.12 g, 91%. ^1^H-NMR (DMSO-*d*_6_) δ 8.62–8.39 (m, 2H), 8.26 (d, *J* = 7.2 Hz, 1H), 8.17 (d, *J* = 7.5 Hz, 1H), 7.95 (t, *J* = 7.4 Hz, 1H), 6.87 (s, 1H), 4.10 (s, 2H), 3.25 (d, *J* = 4.1 Hz, 2H), 1.19 (s, 9H). ^13^C-NMR (DMSO-*d*_6_) δ 163.05, 163.00, 155.77, 132.36, 131.38, 131.25, 130.76, 129.71, 128.83, 128.71, 128.39, 122.99, 122.23, 77.46, 37.63, 28.04. LC-MS (ESI): *m*/*z* 319.21 [M − Boc + H]^+^ (100%). Analytical HPLC: 89% purity (254 nm).

#### 3.2.2. *tert-Butyl 2-(1,3-dioxo-6-((trimethylsilyl)ethynyl)-1H-benzo[de]-isoquinolin-2(3H)-yl)ethylcarbamate* (**2**)

Compound 1 (4.00 g, 9.54 mmol), TMS acetylene (1.12 g, 1.49 mL, 11.5 mmol), Pd(PPh_3_)_2_Cl_2_ (334 mg, 0.48 mmol), CuI (182 mg, 0.95 mmol) and Et_3_N (2.90 g, 4.00 mL, 28.6 mmol) were all dissolved in dry THF (100 mL) and stirred at RT for 2 h under a N_2_ atmosphere. The solution was combined with H_2_O (100 mL) and extracted with dichoromethane (DCM) (3 × 100 mL). The combined extracts were dried over MgSO_4_ and evaporated *in vacuo* to produce a dark brown-black solid, which was subjected to silica gel chromatography (10% EtOAc in DCM,) to yield the product as a pale yellow solid (*R*_f_ = 0.6). Yield: 1.99 g, 48%. ^1^H-NMR (DMSO-*d*_6_) δ 8.52 (dd, *J* = 13.6, 7.8 Hz, 2H), 8.39 (d, *J* = 7.6 Hz, 1H), 7.91 (t, *J* = 8.0 Hz, 2H), 6.80 (t, *J* = 6.2 Hz, 1H), 4.13 (t, *J* = 5.8 Hz, 2H), 3.27 (dd, *J* = 11.6, 5.9 Hz, 2H), 1.21 (s, 9H), 0.34 (s, 9H). ^13^C-NMR (DMSO-*d*_6_) δ 163.20, 162.91, 155.67, 131.27, 131.01, 130.93, 130.85, 129.53, 127.86, 127.34, 125.68, 122.81, 122.39, 104.51, 101.07, 77.32, 37.63, 27.97, −0.37. LC-MS (ESI): *m*/*z* 337.11 [M − Boc + H]^+^ (100%). Analytical HPLC: 94% purity (254 nm).

#### 3.2.3. *2-(2-Aminoethyl)-6-((trimethylsilyl)ethynyl)-1H-benzo[de]isoquinoline-1,3(2H)-dione* (**3**)

Compound 2 (2.00 g, 4.58 mmol) was stirred in a 1:4 (*v*/*v*) mixture of DCM and TFA at RT for 4 h. The DCM and the bulk of the TFA were then removed *in vacuo*, leaving a thick orange liquid. Upon the addition of H_2_O (50 mL), a yellow precipitate formed, which was collected via vacuum filtration, washed several times with cold H_2_O and dried in a vacuum desiccator overnight. Yield: 1.48 g, 96%. ^1^H-NMR (400 MHz, DMSO-*d*_6_) δ 8.50 (ddd, *J* = 8.3, 7.8, 1.0 Hz, 2H), 8.36 (d, *J* = 7.6 Hz, 1H), 7.95 (dt, *J* = 7.4, 4.0 Hz, 2H), 4.30 (t, *J* = 5.8 Hz, 2H), 3.26–3.06 (m, 2H), 0.36 (s, 9H). ^13^C-NMR (101 MHz, DMSO-*d*_6_) δ 163.70, 163.41, 131.55, 131.32, 131.13, 130.90, 129.80, 128.34, 127.31, 125.79, 122.78, 122.37, 105.07, 101.16, 37.61, 37.51, −0.25. LC-MS (ESI): *m*/*z* 337.10 [M + H]^+^. Analytical HPLC: 91% purity (254 nm).

#### 3.2.4. *2-Bromo-N-(2-(1,3-dioxo-6-((trimethylsilyl)ethynyl)-1H-benzo[de]isoquinolin-2(3H)-yl)ethyl)acetamide* (**4**)

Compound 3 (1.50 g, 4.46 mmol), bromoacetyl bromide (1.80 g, 777 μL, 8.92 mmol) and Na_2_CO_3_ (1.42 g, 13.4 mmol) were dissolved in acetone (50 mL) and stirred at RT for 2 h. The solvent was removed *in vacuo* to produce a brown solid. Purification via silica gel chromatography (20% EtOAc in petroleum spirits) yielded the product as a pale yellow solid (*R*_f_ = 0.1). Yield: 1.21 g, 59%. ^1^H-NMR (DMSO-*d*_6_) δ 8.55–8.46 (m, 2H), 8.36 (dd, *J* = 7.6, 3.2 Hz, 1H), 7.98–7.91 (m, 2H), 4.13 (t, *J* = 6.3 Hz, 2H), 3.71 (d, *J* = 14.0 Hz, 2H), 3.54–3.34 (m, 2H), 0.35 (s, 9H). ^13^C-NMR (DMSO-*d*_6_) δ 131.63, 131.45, 131.23, 130.12, 128.67, 127.62, 125.90, 125.81, 123.20, 123.10, 122.82, 122.70, 105.20, 105.12, 101.53, 61.66, 37.26, 36.67, 29.64, 0.06. LC-MS (ESI): *m*/*z* 457.00 (100%) [M + H]^+^. Analytical HPLC: 97% purity (254 nm).

#### 3.2.5. *tri-tert-Butyl 2,2′,2″-(10-(2-((2-(1,3-Dioxo-6-((trimethylsilyl)ethyn-yl)-1H-benzo[de]isoquinolin-2(3H)-yl)ethyl)amino)-2-oxoethyl)-1,4,7,10-tetraazacyclododecane-1,4,7-triyl)triacetate* (**5**)

Compound 4 (350 mg, 0.76 mmol), ^t^Bu_3_DO_3_A.HBr (414 mg, 0.70 mmol), and DIPEA (180 mg, 242 µL, 1.39 mmol) were dissolved in ACN (50 mL) and the mixture refluxed overnight. The solvent was removed *in vacuo* and the residue subjected to silica gel chromatography (5% MeOH in DCM) to isolate the product as a glassy yellow solid (*R*_f_ = 0.1). Yield: 460 mg, 81%. ^1^H-NMR (CDCl_3_) δ 8.56 (dd, *J* = 8.4, 1.1 Hz, 1H), 8.53 (dd, *J* = 7.3, 1.1 Hz, 1H), 8.41 (d, *J* = 7.6 Hz, 1H), 7.81 (d, *J* = 7.6 Hz, 1H), 7.76 (dd, *J* = 8.3, 7.4 Hz, 1H), 4.37–1.97 (broad set of overlapping signals, 28H), 1.42 (d, *J* = 2.9 Hz, 27H), 0.33 (s, 9H). ^13^C-NMR (CDCl_3_) δ 172.51, 172.19, 164.34, 164.02, 132.61, 131.85, 131.74, 131.18, 130.49, 128.00, 127.61, 127.46, 122.75, 122.16, 105.53, 101.26, 81.86, 81.81, 56.49, 55.76, 54.23, 42.60, 39.61, 38.36, 28.24, 28.06, 27.99, −0.08. LC-MS (ESI): *m*/*z* 446.47 [M + 2H]^2+^ (100%). Analytical HPLC: 93% purity (254 nm).

#### 3.2.6. *2,2′,2″-(10-(2-((2-(6-Ethynyl-1,3-dioxo-1H-benzo[de]isoquinolin-2(3H)-yl)ethyl)amino)-2-oxoethyl)-1,4,7,10-tetraazacyclododecane-1,4,7-triyl)triacetic acid* (L)

Compound 5 (320 mg, 0.36 mmol) and KF (42 mg, 0.72 mmol) were dissolved in 1:1 (*v*/*v*) mixture of H_2_O and ACN (10 mL) and the solution stirred for 3 h at RT. The solvent was removed *in vacuo* and the remaining residue dissolved in a 1:1 (*v*/*v*) mixture of TFA and DCM and stirred O/N at RT. After removal of the TFA and DCM using a nitrogen stream, the crude product was purified by preparative HPLC. Lypholisation yielded a fluffy orange solid. Yield: 167 mg, 71%. ^1^H-NMR (CD_3_OD) δ 8.73 (dd, *J* = 8.4, 1.1 Hz, 1H), 8.62 (dd, *J* = 7.3, 1.1 Hz, 1H), 8.52 (d, *J* = 7.6 Hz, 1H), 8.00 (d, *J* = 7.6 Hz, 1H), 7.93 (dd, *J* = 8.4, 7.4 Hz, 1H), 4.42 (s, 1H), 4.49–3.00 (broad set of overlapping signals, 28H). ^13^C-NMR (CD_3_OD) δ 164.40, 164.08, 132.07, 131.78, 131.39, 130.00, 127.79, 127.69, 126.68, 122.65, 122.31, 118.15, 115.24, 39.21, 37.74. HRMS (ESI): *m*/*z* calc’d for [M + H]^+^, M = C_32_H_38_N_6_O_9_: 651.2779, found: 651.2773. Analytical HPLC: 96% purity (254 nm).

#### 3.2.7. Gd-L

Ligand L (38 mg, 47 μmol) and Gd(OAc)_3_ (39 mg, 120 μmol) were dissolved in MilliQ H_2_O (5 mL) and the pH adjusted to *ca.* 7 with 2 M NaOH. The resulting solution was stirred at 70 °C for 2 h, after which time LC-MS showed complete complexation. The product was purified via preparative HPLC and isolated as a fluffy orange solid following lyophilisation. Yield: 20 mg, 53%. Anal. Calc’d for C_32_H_35_N_6_O_9_Gd·6H_2_O: C, 42.10; H, 5.19; N, 9.20. Found: C, 42.09; H, 5.06; N, 9.11. HRMS (ESI): *m*/*z* calc’d for [M + H]^+^, M = C_32_H_35_N_6_O_9_Gd: 806.1785, found: 806.1779. Analytical HPLC: 96% purity (254 nm).

#### 3.2.8. “Clicked” Gd-L

Ligand L (38 mg, 47 μmol) and Gd(OAc)_3_ (39 mg, 120 μmol) were dissolved in MilliQ H_2_O (5 mL) and pH adjusted to *ca.* 7 with 2 M NaOH. The resulting solution was stirred at 70 °C for 2 h. After this time, the pH of the solution was readjusted to *ca.* 7, then 3-azidopropan-1-ol (9.6 mg, 95 μmol), sodium ascorbate (18.7 mg, 95 μmol), CuSO_4_ (0.7 mg, 5 μmol) and THPTA (4.3 mg, 10 μmol) added and the mixture stirred at RT O/N. The product was purified by preparative HLPC and isolated as a fluffy orange solid after lyophilisation. Yield: 20 mg, 47%. Anal. Calc’d for C_35_H_42_N_9_O_10_Gd·6H_2_O: C, 41.45; H, 5.37; N, 12.43. Found: C, 41.82; H, 5.27; N, 12.36. HRMS (ESI): *m*/*z* calc’d for [M + H]^+^ M = C_35_H_42_N_9_O_10_Gd: 907.2374, found: 907.2413. Analytical HPLC: 96% purity (254 nm).

#### 3.2.9. Peptide Conjugation

To a solution of Myr-Lys(N_3_)-YGRKKRRQRRR [55] (10 mg, 5.2 µmol) in MilliQ water (5 mL) was added Gd-L (10 mg, 12 µmol), followed by CuSO_4_ (0.8 mg, 5 µmol), THPTA (4.5 mg, 10 µmol) and sodium ascorbate (3.1 mg, 16 µmol). The pH was then adjusted to 6–7 with 1 M HCl and the mixture stirred O/N at RT. Peptide LC-MS analysis showed near-complete consumption of starting peptide after this time. The product was isolated as a white solid following preparative HPLC and lyophilisation. Peptide LC-MS (ESI): *m*/*z* 986.95 [M + 2TFA + H]^3+^, 90% purity (214 nm).

### 3.3. Quantum Yield Determinations

Complexes were prepared at a range on concentrations in a 100 mM HEPES buffer at pH 7.4, and absorbance and fluorescence emission spectra recorded. Quantum yields (Φ) were then determined, using quinine sulphate in 0.1 M sulfuric acid as the reference compound (Φ = 54%) [80], according to the following equation:

Φ_X_ = Φ_ST_ × (Grad_x_/Grad_ST_) × (Ƞ_X_/ Ƞ_ST_)^2^

where Φ is the quantum yield, X and ST denote the sample and reference, respectively, Grad is the gradient of the integrated fluorescence *vs.* absorbance plot, and Ƞ represents the refractive index of the solvent.

### 3.4. Cell Culture

Tongue squamous carcinoma cells (CAL-33) were cultured in phenol red-free RPMI medium (Gibco) supplemented with 10% fetal calf serum (FCS, Gibco), 1% glutamax (Gibco), 100 U penicillin mL^−1^, 100 μg streptomycin mL^−1^ at 37 °C and 6% CO_2_.

### 3.5. Cytotoxicity Studies

Cytotoxicity studies of the effect of irradiation on CAL-33 cells treated with Gd-L, “clicked” Gd-L and the Gd-L-Tat peptide conjugate were performed via a fluorimetric cell viability assay using resazurin (Promocell GmbH). Briefly, one day before treatment, cells were plated in triplicate into 96-well plates at a density of 4 × 10^3^ cells·well^−1^ in 100 μL of medium. After addition of increasing concentrations of the test compound, cells were incubated for 4 h, then the medium was replaced with fresh medium devoid of compound. The plates were irradiated for 10 min at 350 nm (2.58 J·cm^−2^) in a Rayomet Chamber Reactor. Upon further incubation at 37 °C in 6% CO_2_ for 44 h, the medium was removed and complete medium (100 μL) containing resazurin (0.2 mg·mL^−1^ final concentration) was added. After 4 h of incubation at 37 °C in 6% CO_2_, the fluorescence of the intensely red fluorescent resorufin product was quantified using a SpectraMax M5 microplate reader (excitation: 540 nm; emission: 590 nm).

### 3.6. Cellular Uptake Studies

Cellular uptake and localisation of Gd-L, “clicked” Gd-L and the Gd-L-Tat peptide conjugate was assessed by confocal fluorescence microscopy. CAL-33 cells were grown on 18-mm Menzel-gläser coverslips at a density of 2 × 10^5^ cells·mL^−1^ and incubated for 4 h with one of the compounds (100 μM). Cells were fixed in 4% formaldehyde in phosphate-buffered saline (PBS), washed with PBS and H_2_O, and mounted on slides for viewing. Fixed cells were viewed on a CLSM Leica SP5 microscope (Heerbrugg, Switzerland), with the compounds visualised using the hybrid 1 red wavelength selection on the microscope (excitation: 405 nm; emission: 600−800 nm). It should be noted that there is the possibility that fixation may change the cellular localisation of fluorescent molecules as they may be washed out or redistributed.

## 4. Conclusions

In conclusion, we have developed a new Gd(III)-DOTA-naphthalimide complex that can be readily conjugated to azide-bearing molecules via the Cu(I)-catalysed click reaction to introduce a fluorescent and photo-cytotoxic label. Whilst the photo-physical properties of the naphthalimide group are not optimal for PDT, the complex represents a useful prototype building block for the facile construction of new theranostic agents for combined PDT and optical imaging. By virtue of the Gd centre, the complex could also serve as an effective MRI contrast agent if incorporated into appropriate *in vivo* imaging agent designs. Future work will include measurement of the proton nuclear magnetic relaxation dispersion of the “clicked” Gd-L complex to confirm this capability. Since MRI is a very insensitive technique, it will also be important to establish conjugate designs that are able to deliver sufficient concentrations of Gd-L to desired sites (e.g., diseased tissue) to provide adequate image contrast *in vivo*. 

## Supplementary Materials

The following are available online at http://www.mdpi.com/1420-3049/21/2/194/s1. NMR spectra of reported compounds, LC traces for final ligand, Gd(III) complexes and peptide conjugate.

## Abbreviations

The following abbreviations are used in this manuscript:

ACN: acetonitrile
BOC: *tert*-butoxycarbonyl
^t^Bu_3_DO3A: *tert*-butyl-protected 1,4,7,10-tetraazacyclodocane-1,4,7,10-tetraacetic acid
d: doublet
dd: doublet of doublets
ddd: doublet of doublet of doublets
dt: doublet of triplets
DCM: dichloromethane
DIC: differential interference contrast
DIPEA: *N,N*-diisopropylethylamine
DMF: *N,N*-dimethylformamide
DOTA: 1,4,7,10-tetraazacyclodocane-1,4,7,10-tetraacetic acid
ESI: electrospray ionization
HEPES: 4-(2-hydroxyethyl)-1-piperazineethanesulfonic acid
HPLC: high-performance liquid chromatography
HRMS: high-resolution mass spectrometry
IC_50_: half maximal inhibitory concentration
J: coupling constant
LC-MS: liquid chromatography-mass spectrometry
m: multiplet
MRI: magnetic resonance imaging
NMR: nuclear magnetic resonance NMR
O/N: overnight
PBS: phosphate-buffered saline
PDT: photo-dynamic therapy
PI: photo-toxic index
RT: room temperature
*R*_f_: retention factor
s: singlet
SPAAC: strain-promoted azide-alkyne cycloaddition
t: triplet
TAT: trans-activator of transcription
THF: tetrahydrofuran
TFA: trifluoroacetic acid
THPTA: *tris*(3-hydroxypropyl-triazolylmethyl)amine
TLC: thin layer chromatography
TMS: trimethylsilyl
UV-A: ultraviolet A radiation
Φ: quantum yield
