# Supplementary material for: Cellular Uptake and Photo-Cytotoxicity of a Gadolinium(III)-DOTA-Naphthalimide Complex “Clicked” to a Lipidated Tat Peptide
Source: Molecules. 2016 Feb 5;21(2):194. doi: 10.3390/molecules21020194 (PMC6273236; doi:10.3390/molecules21020194)
Supplement: Supplementary file 1 [file molecules-21-00194-s001.pdf]

Supplementary Information

Cellular Uptake and Photo-Cytotoxicity of a Gadolinium(III)-DOTA-Naphthalimide Complex “Clicked” to a Lipidated Tat Peptide

William I. O’Malley, Riccardo Rubbiani, Margaret L. Aulsebrook, Michael R. Grace, Leone Spiccia, Kellie L. Tuck, Gilles Gasser, and Bim Graham

Contents **Page(s)**

NMR spectra of reported compounds 2–7

LC traces for final ligand and Gd(III) complexes 8

LC trace for peptide conjugate 9

NMR Spectra of Reported Compounds

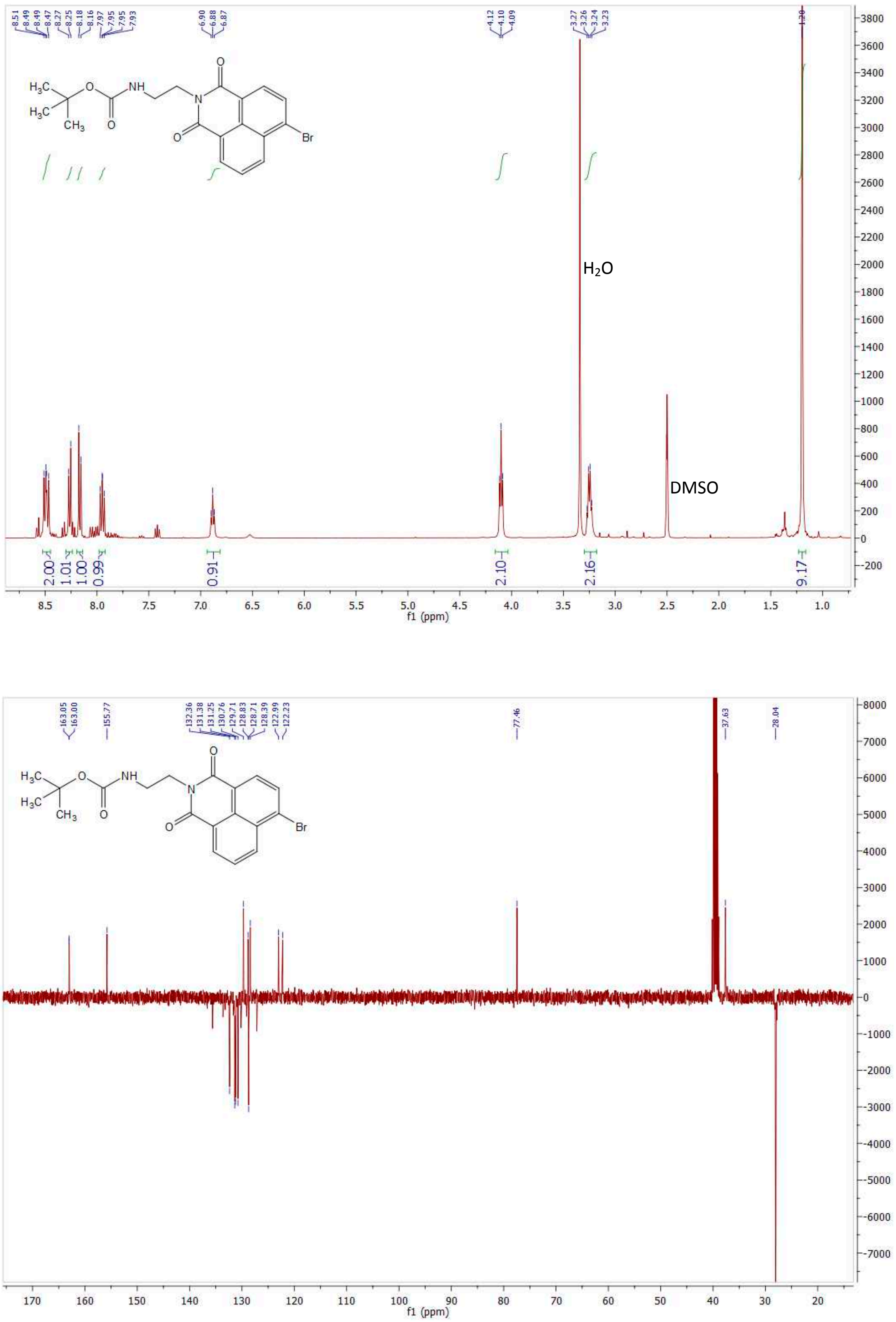

Figure S1. ^{1}H- and ^{13}C-NMR spectra of compound 1 in DMSO-d_6.

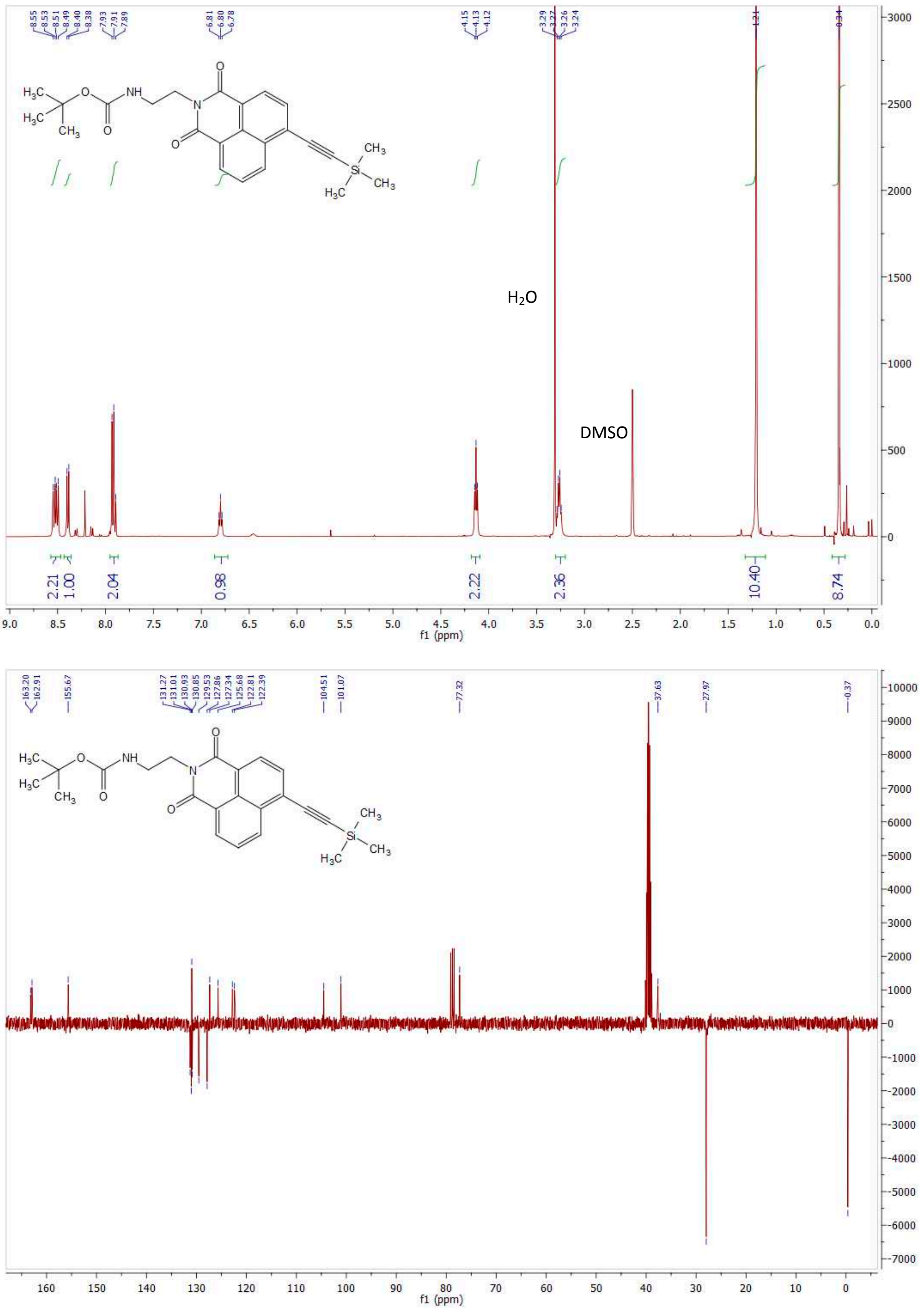

Figure S2. ^{1}H- and ^{13}C-NMR spectra of compound 2 in DMSO-d_6.

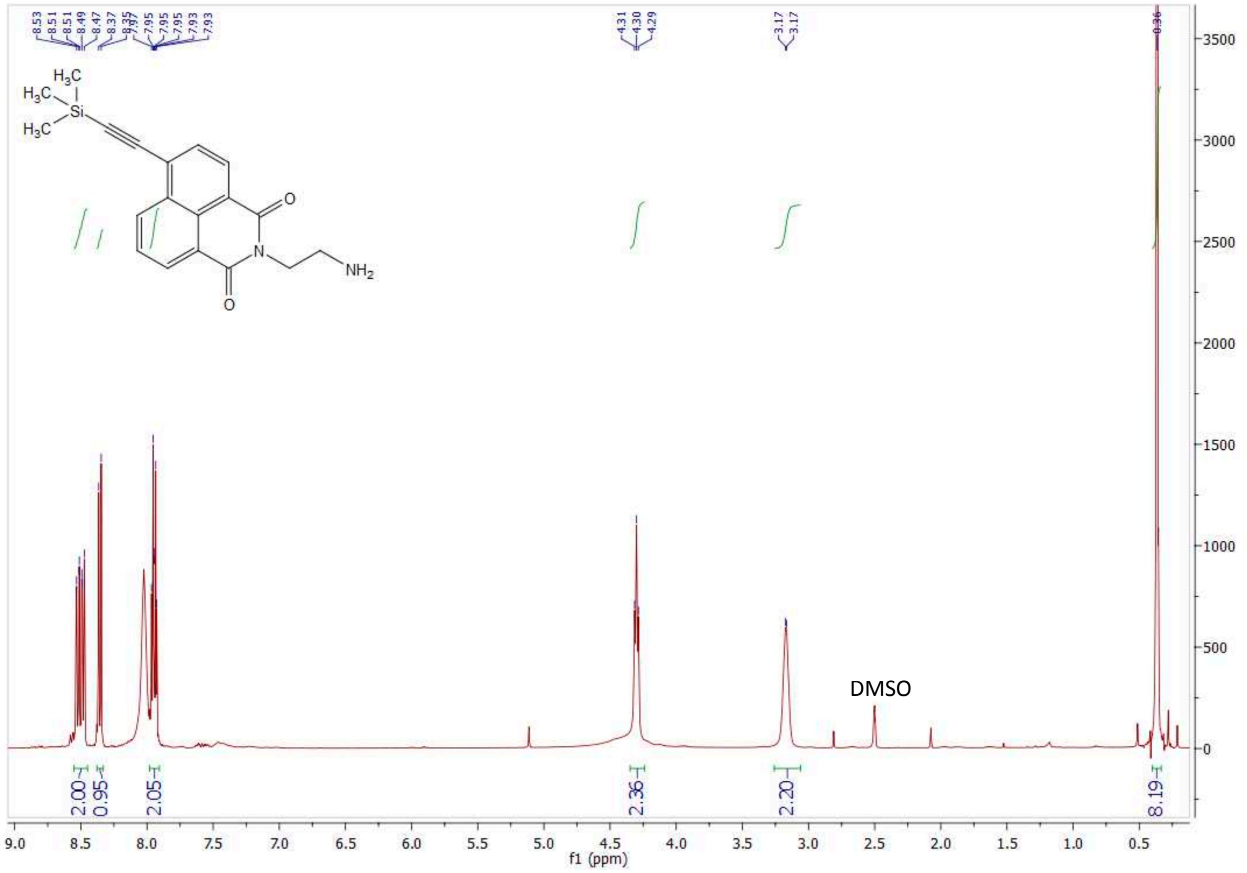

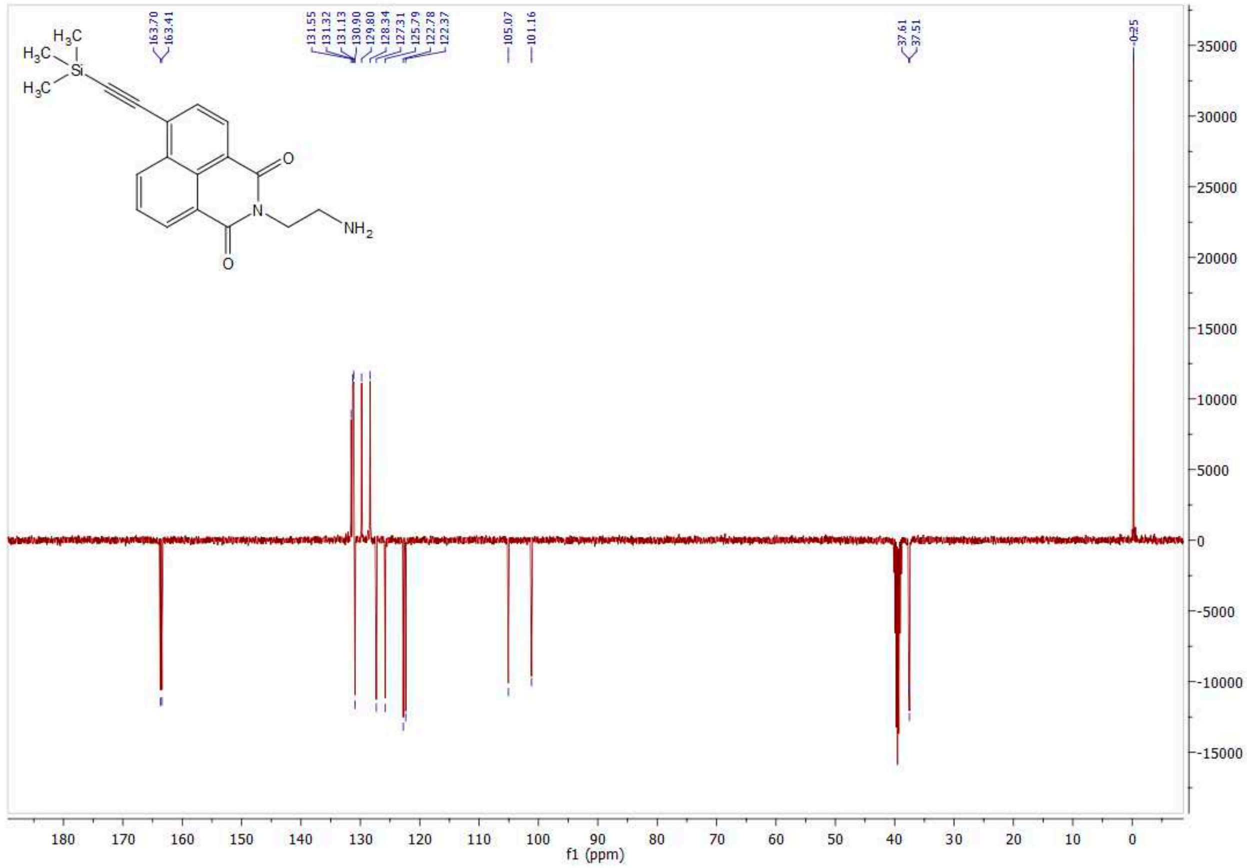

Figure S3. ^{1}H- and ^{13}C-NMR spectra of compound 3 in DMSO-d_6.

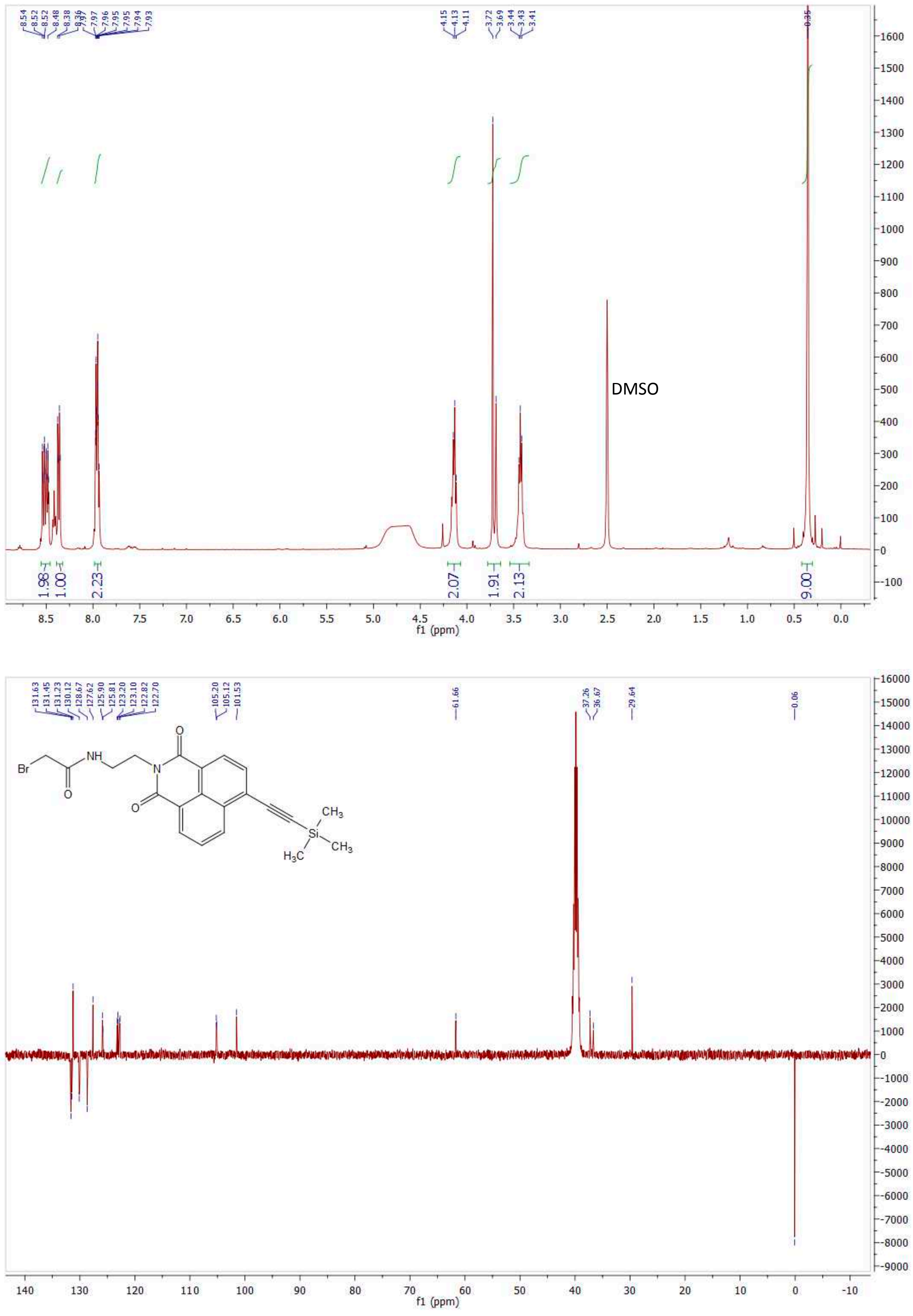

Figure S4. ^{1}H- and ^{13}C-NMR spectra of compound 4 in DMSO-d_6.

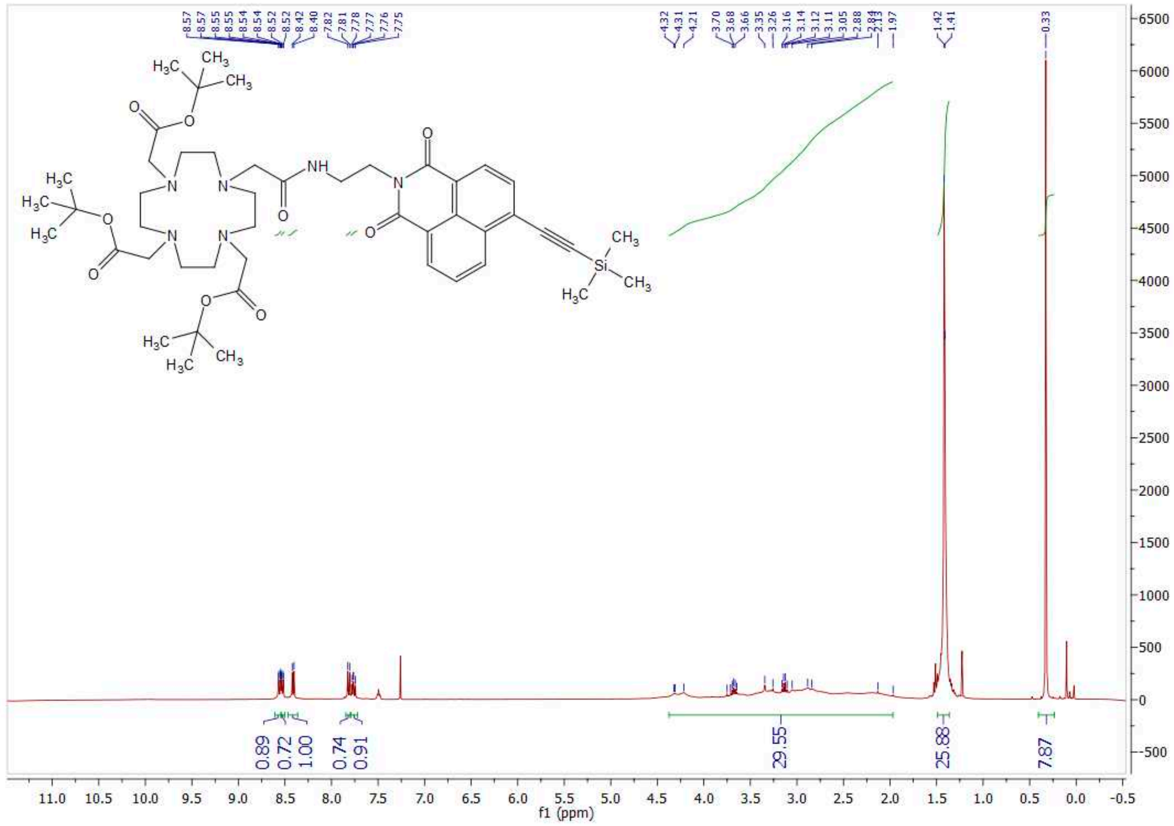

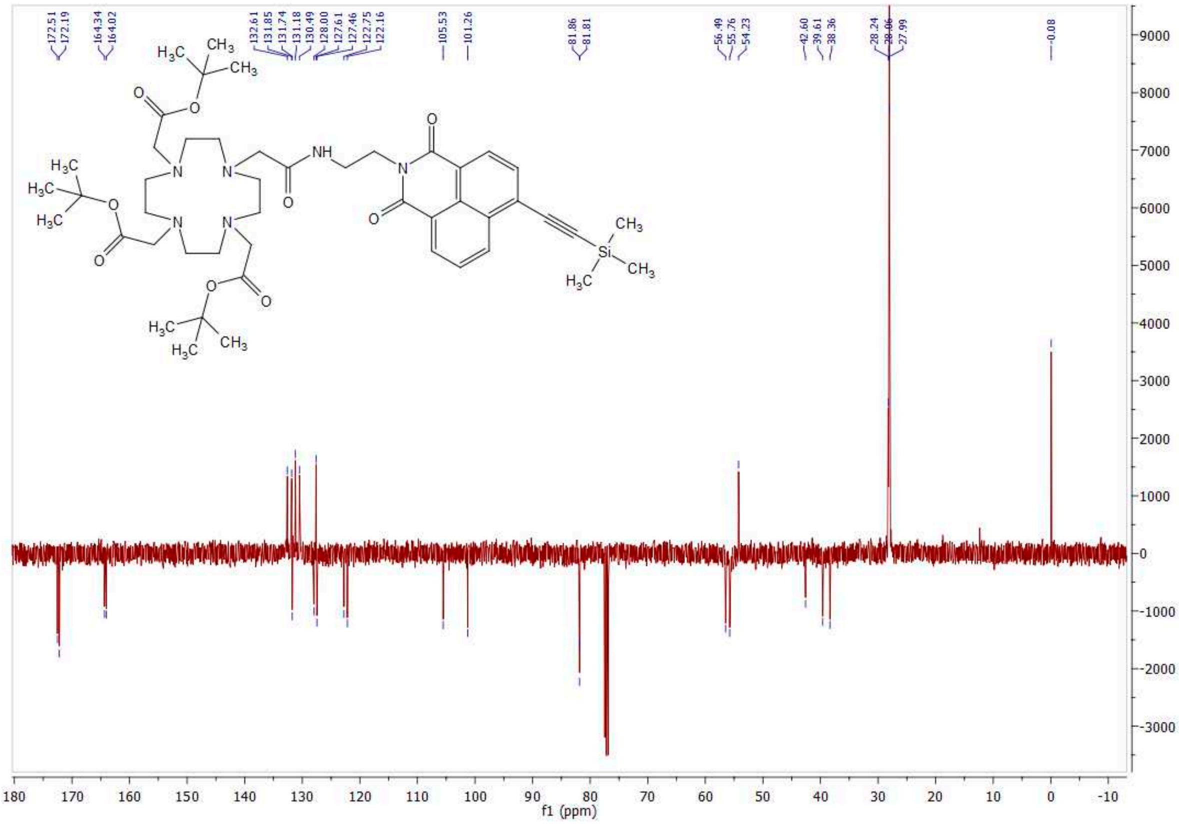

Figure S5. ^{1}H- and ^{13}C-NMR spectra of compound 5 in $CDCl_3$.

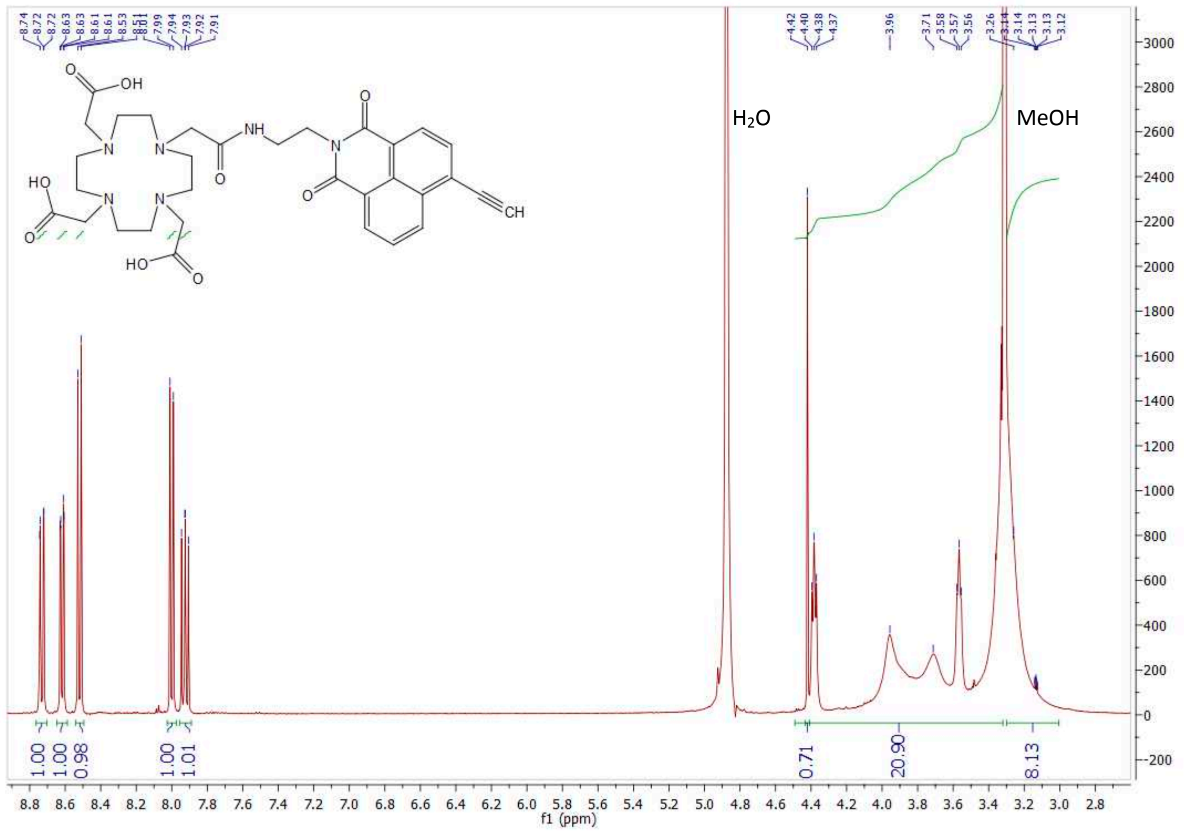

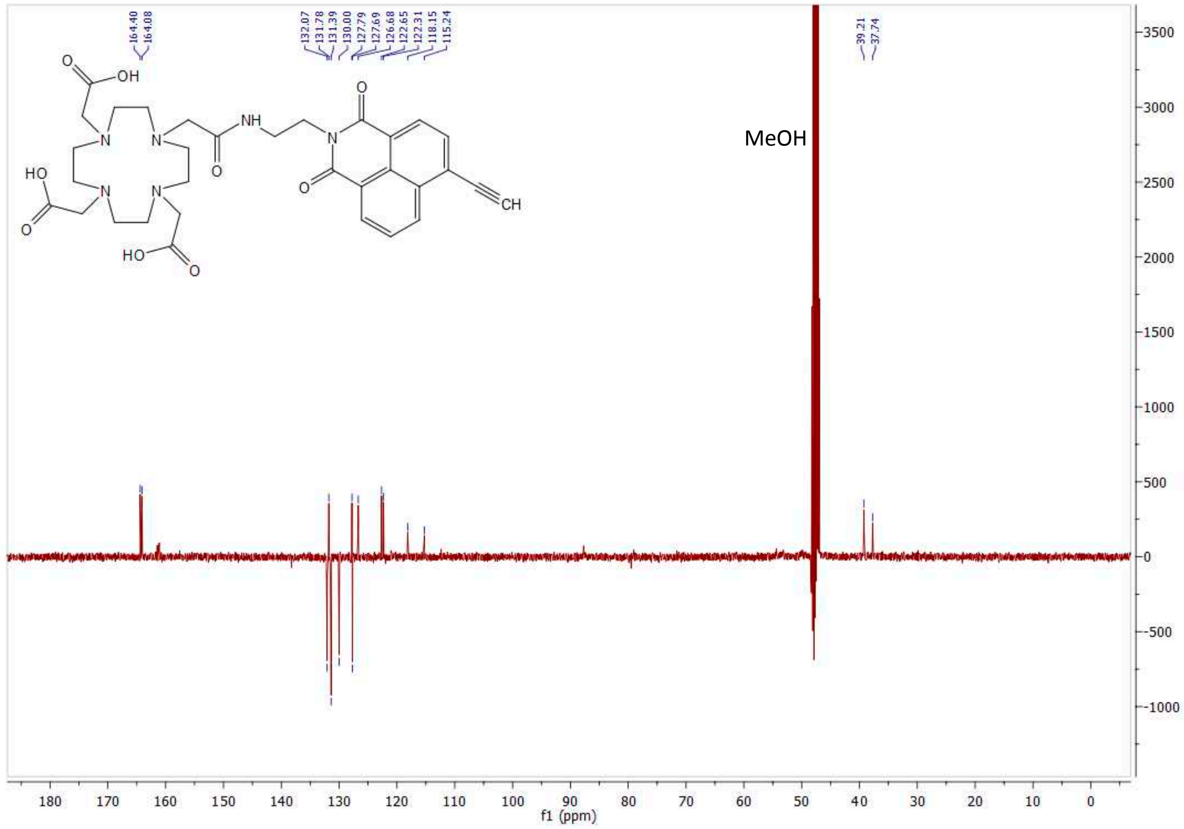

Figure S6. ^{1}H- and ^{13}C-NMR spectra of ligand L in CD_3OD.

LC Traces for Final Ligand and Gd(III) Complexes

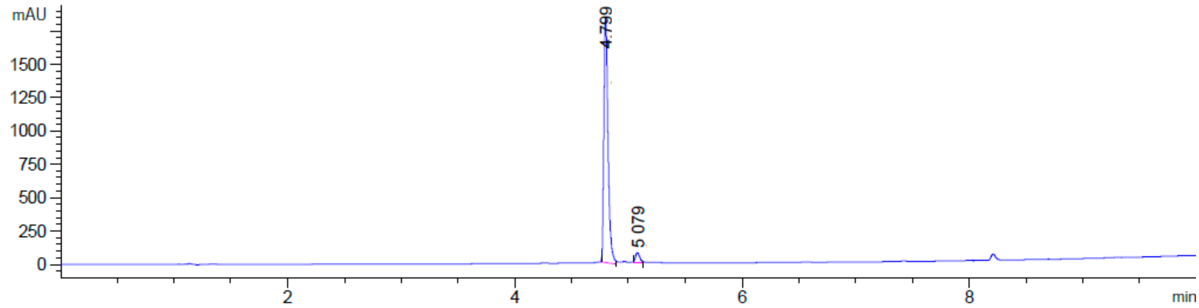

Figure S7. LC trace for ligand L (254nm).

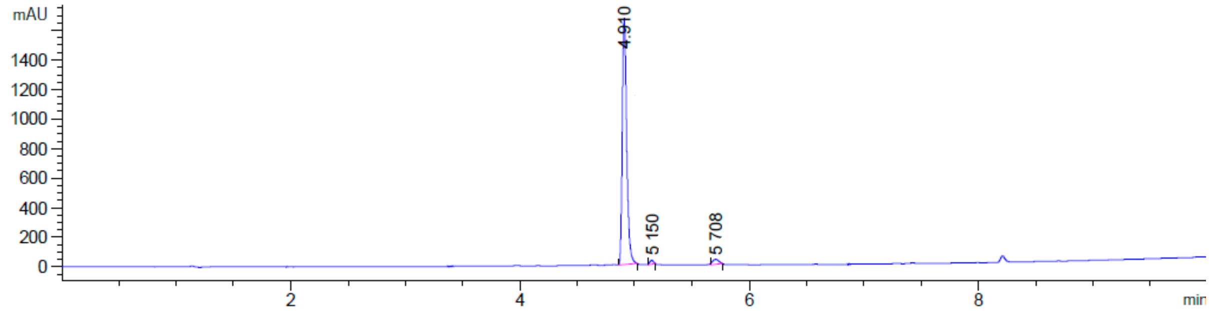

Figure S8. LC trace for Gd-L (254nm).

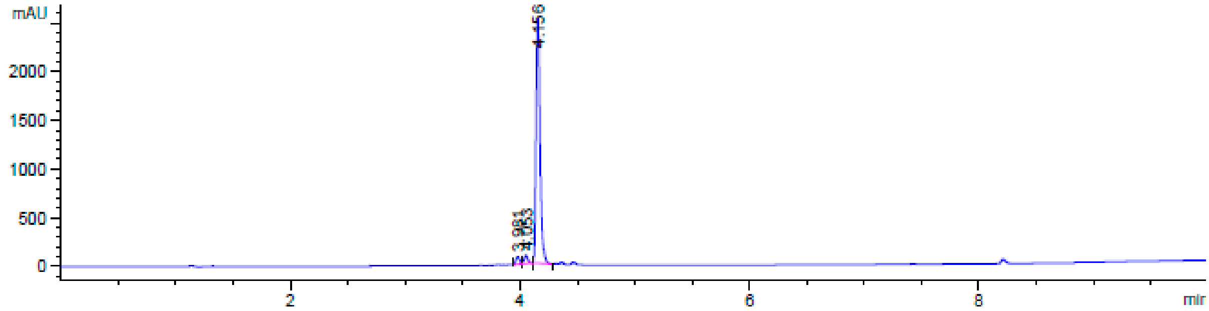

Figure S9. LC trace for “clicked” Gd-L (254nm).

LC Trace for Peptide Conjugate

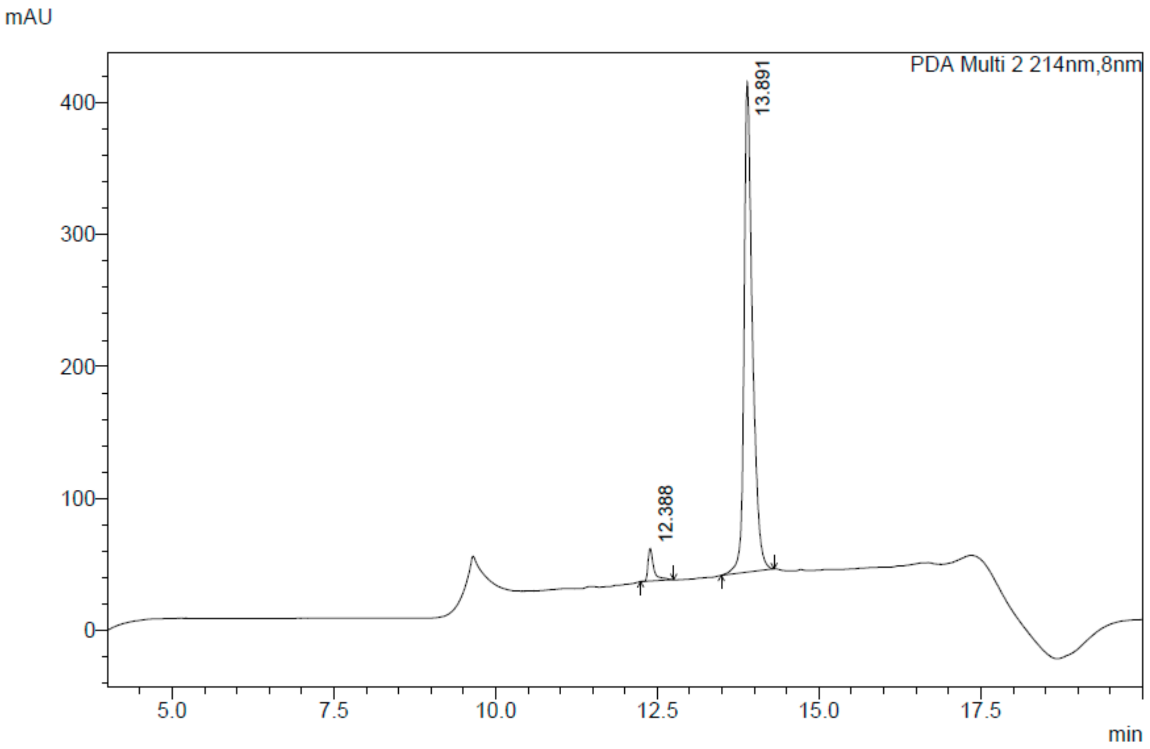

Figure S10. LC trace for Gd-L-Tat peptide conjugate (214 nm).
